# Fewer themes, more stories: shall we consider alternative ways for representing complexity well?

**DOI:** 10.1007/s40037-014-0125-0

**Published:** 2014-06-04

**Authors:** Sayra Cristancho

**Affiliations:** 1 Department of Surgery, Schulich School of Medicine & Dentistry, Western University, London, ON Canada; 2 Centre for Education Research & Education, Schulich School of Medicine & Dentistry, Western University, London, ON Canada

Four years ago, Regehr offered us a challenge—the challenge of openly engaging in conversations around re-examining the goals of medical education research. As described in his ‘It’s NOT rocket science’ paper, for a long time our community has accepted the notion of the physical sciences as the model for *ideal* research [1]. However, as I am sure most of us have experienced, in medical education research things are *hard to control* and therefore *hard to generalize*. Since then it has been gratifying to see the word ‘complexity’ taking prominence in our scientific discourse. However, I am hoping that this word does not become a *cliché* in our community. My main concern, akin to Regehr’s [1] and Lingard et al.’s [2], is how we go about *representing complexity well*.

Frambach et al.’s [3] paper ‘Using activity theory to study cultural complexity in medical education’ served as a welcome opportunity to continue engaging in conversations around representing complexity in medical education research. I am not an expert on cultural studies, but being an immigrant myself with expertise in systems complexity, I couldn’t help but enjoy and relate to the authors’ goals of trying to represent the cultural complexity of problem-based learning. The “Reporting Results” section particularly attracted my attention, especially the authors’ suggestion that ‘researchers could use to their advantage the rich variety in activity theory research of how results can be reported and structured’. I appreciated seeing the authors exerting effort—and precious space in the manuscript!—to reflect on the many ways in which their results could have been presented: themes, stories, visuals. Out of those options, the authors justify the use of themes as the representation method that they ‘felt readers would benefit more from’. I must admit that I felt strangely disappointed by this decision. It led me to wonder, what has made ‘themes’ become the normative representation for the results of studies of complex issues in our community? Other social science disciplines have long ago already established alternative forms of representing complexity. Why are we reluctant to write outside the reductionist box of the physical sciences model, which atomizes and presents results in a linear logic?

As I asked those questions to myself, I began to notice that a few of us may have already taken that path, but likely in an implicit way. Let’s take the option of using stories. We constantly use stories to talk about our experiences. I believe the way we do research is not too different from that. When we interview participants, we most likely hear a story. When we ask participants to draw, they draw stories. Even researchers outside our community have referred to stories as ‘data with soul’ [4]. Observing in the operating room has been a valuable source of stories in my research. One day I decided that in addition to interviewing, I was going to ask surgeons to draw their experiences with very complex operations. After one of those operations, the surgeon and I used the drawing to share with each other our stories about observing [me] and conducting the operation [her], and we found both of us having ‘*aha*!’ moments. From my perspective, this was the realization that surgical complexity goes well beyond the procedural aspects. From her perspective, it was the realization that surgical complexity challenges the dominant cultural expectations of autonomy and invulnerability.

In the scientific writing of medical education research, we are already using stories to portray perspectives through the use of comment boxes. Some authors, as in Frambach et al.’s [3, 5] paper, have done a very good job at effectively using comment boxes to incorporate stories that illustrate particular aspects of their results sections, but it nevertheless seems a constraining sort of approach. We would have us ask ourselves, why comment boxes? What message are we delivering, intentionally or unintentionally, by using comment boxes? Do comment boxes take out some of our most powerful messages and signal that they are ‘optional’, that they are secondary to the main message of the paper? Is it reasonable to suggest hierarchies of reporting methods within an interdisciplinary community like ours? If the goal, as Regehr [1] suggested, is for medical education researchers to become *question-generators* more so than *solution-providers*, we may be better off using stories as a platform to raise our questions. If so, should we consider moving stories from comment boxes to the core of the results sections in our scientific writing?

If we look outside the dissemination venues for medical education research, we begin finding beautifully articulated examples of using stories as platforms to ask questions. Annemarie Mol [6], through her social studies of knowledge practices, shows us such a way to represent complexity. *Cutting surgeons*, *walking patients* is the story of the practices of diagnosis and treatment as lived by characters from three groups: medical professionals, technicians and patients. Rather than reporting individual themes, Mol crafts an overarching narrative story throughout the paper through which the themes become visible and are described. This story intertwines the journeys of the three characters as they try to come to terms with the debate between walking therapy and surgery: ‘In comparing treatments one may ask what these treatments do to a patient’s life as if the treatments themselves were external events. But it all gets a lot more complex once one starts to recognize that those treatments themselves are a (more or less prominent) part of life. And that they imply a certain way of living’. As Mol concludes, ‘In health care, handling complexities, in one way or another, is also an often urgent, practical task. A task that may get squeezed *in between* others.’ And I would add, a task that is shaped by those *in between* stories. Her point in using stories was not to give a ‘normative advice [solution]—as if I knew’, but to attempt to open up those complexities for all involved, as an avenue to foster reflective conversations about current practices.

Stories are just one alternative way of representing research results. The notions of multi-literacies and multimodality that are being taken up in broader education research, expand even further our sense of the range of alternative ways of representing research results. These notions have suggested moving away from a reliance on print toward digital technology, including sound, music, pictures and still and moving images [7, 8]. One could argue that while the use of stories and other non-thematic alternatives to represent complexity in medical education research might be appealing, our dissemination venues are not equipped to offer such alternatives. Shorter manuscript length and shorter presentation timeframes are usually a rationale to leave out stories and opt for thematic descriptions. I am not suggesting a *boycott* of the norms and practices of how we disseminate our medical education research. What I do suggest, though, is engaging in conversations about how we could consider creative ways of reporting results within those norms and practices.

Frambach et al.’s results are certainly intriguing as they highlight the key cultural themes that complicated and shaped PBL learning across three medical schools (e.g., group relations, ‘face’, hierarchy, tradition, uncertainty, achievement and competition). I was however left wondering whether reporting them differently could have made an even stronger case for the complex interrelatedness among those themes. While I am not intending to critique the authors’ decisions for reporting findings, as a reader I would have been even more excited to see those findings presented in a manner that did not constrain their evocative power, such as unique stories from participants. My intention in writing this commentary was therefore to build on the opportunity provided by Frambach et al.’s paper [3] to expand Regehr’s [1] and Lingard et al.’s [2] conversation on *representing complexity well* by continuing to ask more questions. Shall we consider alternative forms of reporting results when it comes to issues of complexity? What implications would that carry for the ways we do research in medical education? What is to be learned and what should we be cautious about? Answers to these questions are not straightforward but at minimum I hope these questions will keep the conversation going.
